# Strategies for community-sourced biocuration in bioinformatics: a case study on MIBiG 4.0

**DOI:** 10.1093/bib/bbaf659

**Published:** 2025-12-11

**Authors:** Kai Blin, Catarina Loureiro, Nico L L Louwen, Jorge C Navarro-Muñoz, Hans Gerstmans, Serina L Robinson, Adriano Rutz, Zachary L Reitz, Drew T Doering, Justin J J van der Hooft, Tilmann Weber, Marnix H Medema, Mitja M Zdouc

**Affiliations:** The Novo Nordisk Foundation Center for Biosustainability, Technical University of Denmark, Søltofts Plads, Building 220, Kongens Lyngby 2800, Denmark; Bioinformatics Group, Wageningen University & Research, Droevendaalsesteeg 1, Wageningen, 6708 PB, the Netherlands; Bioinformatics Group, Wageningen University & Research, Droevendaalsesteeg 1, Wageningen, 6708 PB, the Netherlands; Bioinformatics Group, Wageningen University & Research, Droevendaalsesteeg 1, Wageningen, 6708 PB, the Netherlands; VIB-KU Leuven Center for Microbiology, Flanders Institute for Biotechnology, Kasteelpark Arenberg 31, Leuven 3001, Belgium; Department of Biology, Laboratory for Biomolecular Discovery & Engineering, KU Leuven, Kasteelpark Arenberg 31, Leuven 3001, Belgium; Department of Biosystems, Biosensors Group, KU Leuven, Willem de Croylaan 42, box 2428, Leuven 3001, Belgium; Department of Environmental Microbiology, Swiss Federal Institute of Aquatic Science and Technology, Ueberlandstrasse 133, Duebendorf 8600, Switzerland; Institute for Molecular Systems Biology, ETH Zürich, Otto-Stern-Weg 3, Zürich 8093, Switzerland; Department of Ecology, Evolution and Marine Biology, University of California, 1169 Biological Sciences II, Santa Barbara, CA 93106, United States; Lawrence Berkeley National Laboratory, US Department of Energy Joint Genome Institute, 1 Cyclotron Road, Berkeley, CA 94720, United States; Bioinformatics Group, Wageningen University & Research, Droevendaalsesteeg 1, Wageningen, 6708 PB, the Netherlands; Department of Biochemistry, University of Johannesburg, C2 Lab Building 224, Kingsway Campus, Cnr University & Kingsway Road, Auckland Park, Johannesburg 2006, South Africa; The Novo Nordisk Foundation Center for Biosustainability, Technical University of Denmark, Søltofts Plads, Building 220, Kongens Lyngby 2800, Denmark; Bioinformatics Group, Wageningen University & Research, Droevendaalsesteeg 1, Wageningen, 6708 PB, the Netherlands; Bioinformatics Group, Wageningen University & Research, Droevendaalsesteeg 1, Wageningen, 6708 PB, the Netherlands

**Keywords:** biocuration, open science, data standards, genome mining, secondary metabolites

## Abstract

Biocuration is essential to transform molecular sequence data into standardized, machine-readable resources. Such curated datasets enable comparative analysis, predictive modeling, and data integration across bioinformatics platforms. While professional biocuration is resource-intensive and usually limited to institutional settings, community-driven approaches can mobilize large-scale annotation of specialized datasets and are more resilient to disruptions in scientific funding. Here, we present a model for community-powered curation applied to the Minimum Information about a Biosynthetic Gene Cluster (MIBiG) repository. Through a framework of workflows for metadata capture, annotation validation, and contributor coordination, the MIBiG 4.0 initiative recruited 267 scientists across 178 institutions from 33 countries, volunteering an estimated 4000 h of work. These efforts expanded the MIBiG repository by 22% and enhanced its usability in downstream molecular data analyses in comparative genomic analyses, natural product discovery, and machine learning applications. We provide strategies and actionable lessons for adopting this model, supporting the sustainability of curated bioinformatics resources central to nucleic acid research and related fields.

## Graphical Abstract

**Graphical Abstract:**
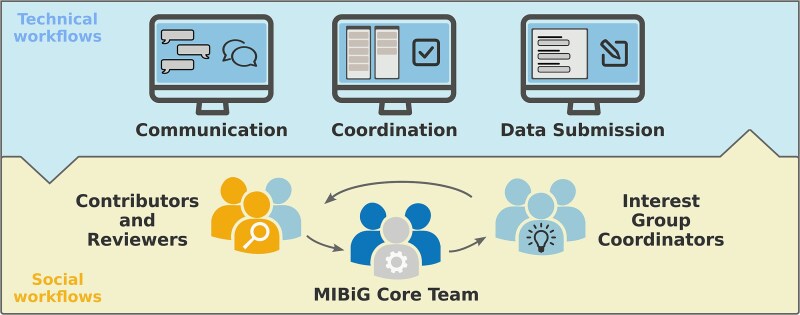

## Introduction

Biological research activities, such as (meta) genome sequencing, generate large quantities of data, but the interpretations of their results are customarily published in narrative form as journal articles. Despite the development of data standards and associated online repositories for several key types of biological data, descriptions of the characterization of various kinds of biological and biochemical entities are still frequently released as heterogeneous, unstructured data files, lacking metadata. Such data formats are challenging to directly interpret with computers, hindering large–scale comparison, reuse, and data integration. Biocuration is the process of transforming these dispersed and unstructured data into structured, computable resources [1]. It involves tasks such as extracting and validating biological information from experimental studies, harmonizing terminology and metadata, and integrating the results into existing knowledge frameworks such as databases or ontologies. Consequently, biocuration serves as a bridge between primary research outputs and machine-readable data, enabling standardized and comprehensive computational analysis, supporting machine-learning applications, and promoting FAIR (Findable, Accessible, Interoperable, and Reusable) data principles [2–5]. Despite its crucial importance to nucleic acid research, biocuration also faces challenges. Performed by highly educated and experienced experts, professional biocuration is resource-intensive and usually limited to institutional bodies [6]. Even though the benefits of biocuration are estimated to be more than 20 times their direct operational costs [1], traditional academic crediting systems (e.g. citation counts, article publications) are rarely applicable to biocuration, reducing its visibility [2]. While text mining and large language models (LLMs) may be used in domains such as sequence annotation and biosynthetic gene cluster (BGC) annotation [7–9], they still require expert oversight and validation and should be considered preprocessing tools rather than autonomous actors [10–14]. As an inherently manual process, biocuration is a major rate-limiting step in the interpretation and subsequent systematization of biological data, directly impacting the availability of high-quality molecular datasets and resources usable in comparative genomics or the training of machine learning algorithms.

To address this bottleneck, crowd-sourced and community-driven biocuration strategies have been developed. For instance, biocuration initiatives centered on education benefit from a large pool of (under) graduate participants and can produce high-quality annotations while supporting learning outcomes, but they are usually limited to a single institution and require substantial initial training and ongoing supervision [15, 16]. Another example is the general-purpose knowledge base Wikidata, which provides a readily available, scalable, and community-driven platform for expert data curation and has been successfully applied to the curation of biosynthetic pathways [17]. However, its underlying data structure and editing conventions require familiarization, and the absence of dedicated data validation and review mechanisms present a potential barrier to Wikidata’s adoption by projects that demand stringent data accuracy. Furthermore, domain-specific content may fall outside Wikidata’s notability guidelines, requiring additional considerations before data deposition. These limitations reduce the practicality of current crowd-sourced biocuration strategies for specialist initiatives that require rigorous data validation and editorial oversight, such as BGC databases, enzyme function annotation, and other molecular data resources. There is a need for platforms that offer a low barrier to entry, effectively use the time and expertise of domain experts, are cross-institutional, and can accommodate a large number of participants. At the same time, such models need to ensure sustainability, governance, and attribution mechanisms, while also producing standardized data outputs that integrate well into computational bioinformatics pipelines [1, 18].

The Minimum Information about a Biosynthetic Gene Cluster (MIBiG) repository [19, 20] is regarded as the gold-standard reference database for BGCs by the natural products community. MIBiG is widely used by bioinformatics tools for genome mining, comparative genomics, and natural product discovery pipelines [21–28]. Since its inception in 2015 [29, 30], MIBiG has been maintained and updated by a small, cross-institutional volunteer team, regularly expanding its data schema and coverage by community-driven annotation hackathons (“Annotathons”) in line with scientific advances [29, 31, 32]. However, these activities were organized mostly on an *ad hoc* basis and encountered difficulties in accommodating a growing number of international participants. During the preparation of the fourth iteration of the MIBiG Annotathons (MIBiG 4.0), we sought to improve our data curation workflow by creating a dedicated organization framework consisting of workflows for data collection and validation, communication, and task coordination, inspired by the Open Data, Open Code, and Open Infrastructure (O3) guidelines [18]. Centered on Annotathon events, this biocuration model enabled the largest MIBiG data curation effort to date, engaging 267 scientists across 178 institutions from 33 countries spanning 20 time zones (New Zealand to California) who contributed an estimated 4000 person-hours to extract and curate literature-derived data [20]. In this *Position* article, we present the biocuration model developed through the MIBiG 4.0 initiative, evaluate its impact on the quality and sustainability of molecular data resources, compare it to selected existing community curation models, and provide recommendations for related efforts seeking to adopt the model. We believe that this biocuration model provides a scalable strategy for generating high-quality, community-driven bioinformatics resources across nucleic acids, protein, metabolite, and integrative omics databases, suitable for adoption by both new and established resources [33–41].

### Preparing foundations: establishing the project framework

**Figure 1:**
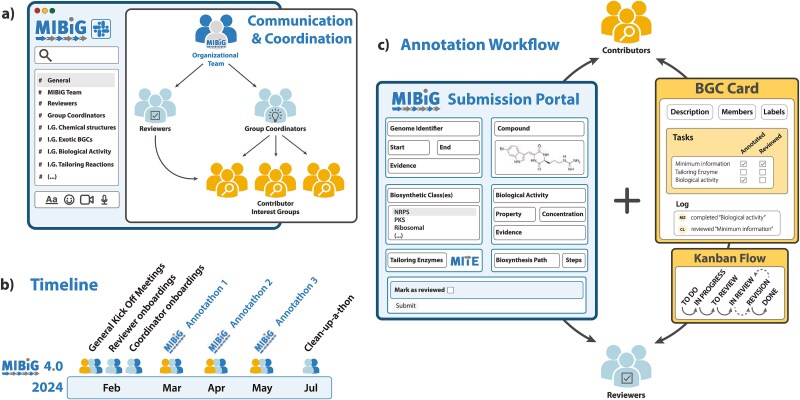
Schematic overview of curation effort. The figure shows the organizational aspects and workflow governing the MIBiG 4.0 curation model. (a) Communication and coordination framework: the MIBiG organizational team directs and supports the interest group coordinators and reviewers, who, in turn, guide and assist contributors with their annotation effort. (b) Timeline of major organizational events leading to, during, and after the MIBiG 4.0 Annotathons: After the Annotathons, an internal “clean-up-a-thon” was held to finalize unfinished entries. (c) General curation workflow: contributors coordinate through Kanban boards, submit data to the MIBiG submission portal, followed by review and approval (or request for changes) by at least one reviewer.

Successful community-driven biocuration projects require a clear organizational framework, with a well-defined governance model to ensure maintenance and sustainability of the resource [18]. For MIBiG 4.0, the existing governing body was expanded to establish a core organizational team with dedicated roles and responsibilities (Fig. 1a): a project officer, infrastructure officer, software engineer, and communications officer. This team was responsible for setting up infrastructure, coordinating the overall curation effort, and implementing editorial oversight, such as decision-making about accommodating edge cases in the curation workflow. During the initial preparation phase, the team established foundational governance elements, guided by a clear project aim: to update the MIBiG repository with the latest knowledge on secondary metabolite BGCs and to establish a review process for submitted data. To support this aim, two new community roles outside the core organizational team were introduced and staffed with volunteer field experts (Fig. 1a): (i) interest group coordinators, who assisted individual contributors (placed in predefined interest groups that connected participants with respect to specific interests, e.g. biosynthesis, chemical structures) within specific areas of expertise, helped resolve domain-specific questions, and facilitated the creation of detailed, high-quality entries; and (ii) reviewers, who evaluated submitted entries, providing feedback where needed to ensure data accuracy and consistency. Finally, a detailed project timeline was created, structured around key milestones including participant recruitment, training sessions, and data collection events (Annotathons), with clearly defined deadlines (Fig. 1b).

### Participant recruitment and onboarding

Successful community curation efforts rely on clear recruitment and onboarding strategies that define the project scope, set contributor expectations, and equip participants with the tools needed to contribute effectively. These strategies must also accommodate diverse backgrounds and establish mechanisms for resolving potential conflicts [18]. In the MIBiG 4.0 initiative, participant mobilization targeted both previous Annotathon contributors and individual domain specialists, complemented by calls for participation on social media, resulting in 398 preregistrations. Follow-up kickoff events (Fig. 1b) introduced potential participants to their roles, familiarized them with the project’s collaborative model, infrastructure, and outlined communication and coordination workflows. The criterion for co-authorship (a time-commitment of at least 6 h of biocuration) was defined, and a code of conduct based on the Contributor Covenant (https://www.contributor-covenant.org/) was established. To support onboarding, we developed comprehensive training resources outlining editorial guidelines, including educational videos and a step-by-step data submission guide [42]. Given the highly international composition of the participant pool, kickoff meetings were scheduled across multiple time zones to ensure accessibility.

### Creating effective communication channels

Effective communication is vital for the success of global, remotely coordinated community efforts. To this end, the MIBiG 4.0 curation model employed a mix of synchronous and asynchronous communication strategies. Formal announcements, including schedules, training materials, and event updates, were distributed synchronously via email to ensure consistent outreach. To foster community building and informal, real-time interactions between participants, an instant messaging application was used. Already utilized in previous Annotathons, a “workspace” of the messaging application Slack (https://mibigannotathons.slack.com) was updated to include interest group-dedicated “channels”, each moderated by one or more interest group coordinators (Fig. 1a). During Annotathon sessions, a live video call using the Zoom software (https://www.zoom.com/) was available to facilitate real-time, face-to-face interaction and support in interest group-specific breakout rooms. Of note, we do not endorse any of the closed-source, commercial tools that were utilized in the MIBiG Annotathons, which were mainly selected due to availability and/or widespread use by participants.

### Infrastructure development and workflow implementation

**Figure 2:**
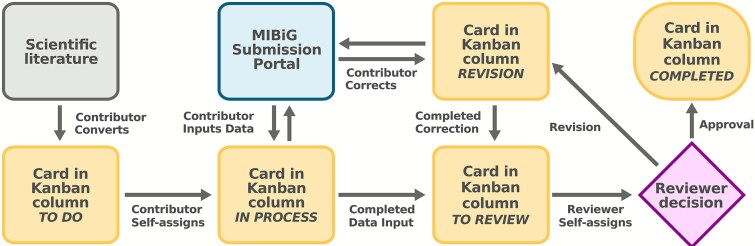
Workflow of the curation process. Starting from scientific literature, contributors create cards on the Kanban board, representing either newly proposed BGC records or issues identified in existing entries. Contributors self-assign to cards and enter data through the MIBiG submission portal. Once a submission is completed, the corresponding card becomes available for review. Reviewers, like contributors, can self-assign to cards and perform reviews via the MIBiG submission portal. Reviewers may request revisions requiring further corrections or approve entries, after which the record is considered complete.

Effective biocuration relies on three key parameters: a defined data curation format, standardized data capture, and effective data validation strategies, which directly shape the workflow design and infrastructure requirements. In the MIBiG repository, BGC entries are stored as JavaScript Object Notation (JSON) text files, defined by a regularly updated bespoke JSON Schema (https://json-schema.org/) data standard [20]. To facilitate interaction with this data model, a dedicated submission portal was created (Fig. 1c). This web server [43] employed a series of forms with built-in data validation, safeguarding against erroneous input. Additionally, the use of persistent identifiers and application programming interfaces (APIs) allowed querying of related resources, such as fetching chemical compound information present in the NPAtlas database. A login system and user privilege management allowed the use of the web portal for both data submission and review. To assess contributor engagement, each edit (a modification submitted through the portal) was recorded, timestamped, and linked to an anonymized contributor ID. Since the time investment per edit could vary considerably, we also gathered self-reported time commitments and other qualitative data through an anonymous post-hackathon questionnaire (Supplementary File 3), complemented by anonymous observation letters from selected participants (Supplementary Information). Around the MIBiG submission portal, a data curation workflow was designed (Fig. 2). To promote autonomous and efficient task coordination, we prepared an online Kanban-style work management system using the free tier version of Trello (https://trello.com). Each phase of the data curation workflow of data collection, review, and revision was represented as columns, with individual entries modeled as cards (Fig. 1c) [20]. This structure provided a transparent overview of task status and availability, thereby facilitating distributed collaboration among remote participants. Contributors and reviewers were able to self-assign tasks and coordinate directly, while a tag-based system supported targeted requests for specialist input without requiring centralized oversight. Doing so, workflow bottlenecks could be promptly detected, and problematic entries flagged for revision, ensuring continuity and consistency throughout the curation process. Before the Annotathons, the Kanban boards were prepopulated with 817 “stub entries”, corresponding to either newly proposed BGC records or identified issues in existing entries (Supplementary File 1).

### The MIBiG curation model resulted in high yield and contributor satisfaction

**Figure 3:**
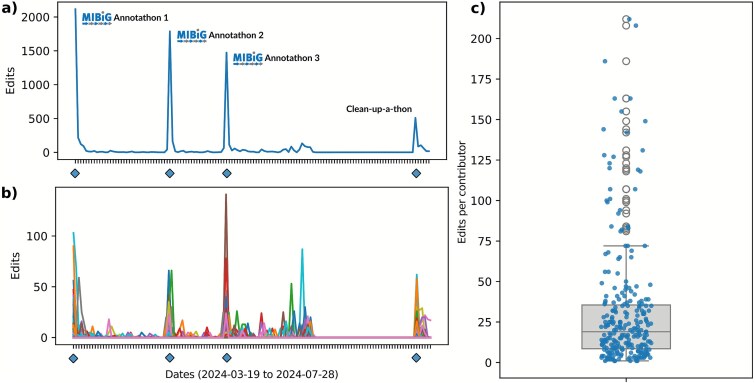
Overview of contributor engagement, measured in edits performed in the MIBiG submission portal. (a) Shows the total count of edits per day (horizontal axis shared with b)), with blue diamonds indicating individual Annotathon dates (two 3-h sessions per day; “clean-up-a-thon” referring to data completion session with a smaller number of participants); (b) breaks down the edit count per day to individual contributors, each assigned a random color; (c) boxplot showing the distribution of edits per contributor (median 19, mean 31.1, SD 37.5), with each blue dot representing an individual contributor in the overlaid strip plot.

In 2024, the MIBiG 4.0 initiative held eight 3-h Annotathon sessions, engaging 267 scientists in data curation and review efforts, over three times the number of participants from the previous edition [20, 32]. In total, contributors performed 8304 edits using the MIBiG submission portal, creating 557 new entries and updating 590 existing ones, growing the MIBiG repository by 22% and expanding nearly 25% of existing entries [20]. Based on self-reported data, this effort represented an estimated total of 4000 volunteer hours (Supplementary File 3). Analysis of activity over time showed that contributor participation was highest during scheduled Annotathon events, with 71% of edits occurring throughout 4 days (Fig. 3a). Additionally, 29% of edits (2417) occurred outside scheduled events (Fig. 3b), indicating that many participants appreciated the possibility to continue working outside the planned Annotathon hours. Edit distribution per participant revealed that a small group of “super-contributors” (10% of participants, Fig. 3c) was responsible for 42% of all edits (Supplementary File 2). This pattern mirrors observations in other community science efforts, such as Wikidata [44], highlighting the importance of recognizing, rewarding, and retaining these individuals for future community initiatives, ideally in leadership and mentorship roles, if possible.

To get insight into participant demographics and assess satisfaction with the MIBiG curation model, we conducted an anonymous exit poll, to which 82 participants (28% of contributors) responded. Additionally, five participants provided detailed qualitative observations (Supplementary Information). The 82 respondents represented a diverse, global community with members across all academic career stages, from (under) graduate students to senior faculty. Generally, satisfaction among respondents was high. 67% identified contributing to a shared effort as their primary motivation (Supplementary Figure S5). Notably, 91.5% indicated they would participate again (Supplementary Fig. S18) and recommend the initiative to others (Supplementary Fig. S19), even though 76.8% were first-time participants (Supplementary Fig. S4).

### Case study

**Figure 4:**
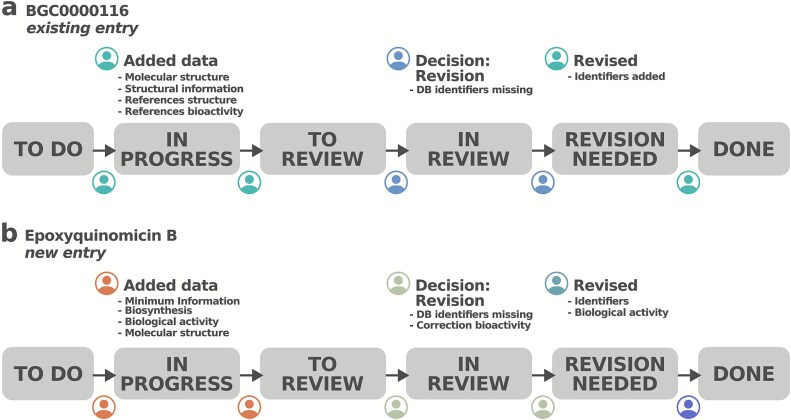
Examples of biocuration within the MIBiG workflow. (a) Improvement of an existing entry, in which the contributor and reviewer followed a traditional peer-review model (Supplementary Fig. S21). (b) Creation of a new entry through the coordinated input of four participants. Notably, a contributor other than the original creator performed the revision, illustrating the collaborative practices fostered in the MIBiG Annotathons (Supplementary Fig. S22).

To gain insight into the social dynamics and interaction patterns that emerged during the MIBiG Annotathons, we examined in detail the provenance and creation history of selected entries. Activities were tracked through Kanban board cards, which also facilitated communication among participants via comments. Two distinct modes of interaction were observed. The first followed a traditional workflow, in which a contributor created an entry that was subsequently peer-reviewed, revised upon request, and corrected by the original contributor (Fig. 4a). In contrast, we also frequently observed a more collaborative pattern, exemplified in Fig. 4b, where multiple contributors jointly refined an entry: contributor 1 initiated the entry, a reviewer suggested revisions, contributor 2 implemented the changes, and a second reviewer performed the final inspection and completion. Although anecdotal, these case studies illustrate the advantages of a Kanban-based coordination framework for managing community-driven, parallel annotation efforts, thereby making use of complementary expertise in an effective manner.

### Systematic comparison with related biocuration models

To explore the transferability of the MIBiG curation model to other communities, we performed a systematic comparison with related collaborative biocuration strategies (Table 1). We investigated the type of collaboration model, its target audience, and participant autonomy, compared the required technical and semantic expertise (knowledge of programs and ontologies, respectively), evaluated the complexity of customizing the provided curation infrastructure, and outlined data quality assurance. The MIBiG 4.0 collaboration model allowed the recruitment of a large number of international experts who performed a high volume of data curation in a relatively short time (eight 3-h sessions) due to adherence to principles described by the Behavioural Insights Team as the EAST framework (Easy, Attractive, Social, Timely, https://www.bi.team/wp-content/uploads/2014/04/BIT-EAST-1.pdf). While attendance in the MIBiG Annotathons presumed expertise in some aspect of NP research, participation was “easy” since no knowledge of data structures, ontologies, or specialist software was required, with graphical user interfaces for data capture (MIBiG submission portal) and coordination (Kanban) appealing to experimentalists. While contribution to the MIBiG database undoubtedly benefited researchers using it as a reference database or as a knowledge base, the MIBIG curation model also presented a tangible and attractive incentive (co-authorship with a subsequent publication) depending on clearly communicated requirements (at least 6 h of curation work). Organizing participation in the form of hackathons introduced a valuable social component, fostering informal collaboration, community-building, and creating a sense of belonging to a collective effort. Additionally, the MIBiG curation model emphasized timeliness, with defined hackathon dates and clear deadlines conveying a sense of urgency. We believe that these aspects can be generalized to other biocuration efforts, motivating the creation of novel resources, and allowing existing systems to expand their efficiency. Continuously running biocuration models could mobilize existing and new participants in hackathon-like events, incentivizing participation with co-authorships on community papers. Models employing technically challenging vocabularies could benefit from dedicated interfaces that facilitate data input, validation, and review. The MIBiG curation model has already inspired the creation of a related resource, the Minimum Information about a Tailoring Enzyme database, which combines a continuous model with regular hackathons and invites all data contributors to be co-authors with its subsequent publication [45]. At the same time, the MIBiG curation model is organizationally demanding, requiring detailed project planning and bespoke infrastructure. Contributor expertise may vary considerably, and the focus on easy-to-use data capture systems over ontologies can impact data quality consistency, which may be addressed with additional participant training. Therefore, related efforts may carefully consider the advantages and disadvantages of the MIBiG curation model and its components, as specified in detail in the next section.

**Table 1:** Systematic comparison between MIBiG 4.0 and other representative community-driven biocuration models.

Name & reference	Description	Collaboration model & participant autonomy	Required technical and semantic expertise	Curation infrastructure setup complexity	Data input control & quality assurance	Transferability of curation model (data, organizational)
**MIBiG 4.0** [20]	Curation of natural product biosynthetic gene clusters	Hackathons recruiting field experts; high autonomy	**Low** (online web portal, forms with auto-fill)	**High** (data submission web server, Kanban board for coordination)	**High** (automated validation, peer review)	**High**
**CACAO** using **GONUTS** software [15]	Curation of gene ontology terms	Education-based competitions recruiting undergraduates; low autonomy	**Medium** (online wiki instance; knowledge of Gene Ontology terms)	**High** (custom wiki instance)	**High** (ontologies, peer review)	**Medium** (university-level, focus on Gene Ontology curation)
**PomBase** using **Canto** software [46, 47]	Knowledge base for fission yeast *Schizosaccharomyces pombe*	Continuous curation recruiting publication authors per email; high autonomy	**Medium** (online annotation workflow; ontology knowledge required)	**Medium** (web server instance can be customized)	**High** (ontologies, peer review, co-curation of contributor and professional curator)	**High** (variable ontologies)
**LOTUS** using **Wikidata** [17, 40]	Curation of natural product provenance (producing organism)	Continuous curation without dedicated recruitment; high autonomy	**High** (knowledge of ontologies; contribution conventions)	**High** (ontology implementation, considerations to adhere to notability guidelines)	**Low** (ontologies available but limited automated checks, no peer review)	**Low**
**Wikipathways** using **GitHub** [35]	Biosynthetic pathway curation	Continuous curation without dedicated recruitment; high autonomy	**High** (extensive training to use PathVisio software)	**Medium** (leveraging free-to-use infrastructure such as GitHub)	**High** (automated checks, peer review)	**High**
**PDBe-KB** using institutional infrastructure [48]	Aggregator of functional annotations for Protein Data Bank	Continuous curation addressing partner resources; low autonomy	**High** (structured JSON files deposited via FTP)	**High** (software stack of private/public FTP, API, webpage)	**High** (defined data exchange format, processing pipeline)	**Low** (complex infrastructure, technical expertise)
**Paired Omics Data Platform** using institutional infrastructure [41]	Reference database for paired genomic and metabolomic data	Initial recruitment of selected experts (high autonomy); currently continuous curation (low autonomy)	**Medium** (online annotation workflow; ontology knowledge required)	**High** (ontology implementation, considerations to adhere to notability guidelines)	**High** (automated checks, peer review)	**Medium**

### Lessons learned in developing a community-driven biocuration framework

The MIBiG 4.0 biocuration model enabled a series of successful Annotathon events, significantly expanding the MIBiG repository as a reference database for bioinformatics applications. The Kanban-style work management system proved to be highly beneficial in guiding efforts and implementing editorial oversight. Pre-populating the Kanban boards with “stub entries” helped focus participants’ attention and facilitated self-directed, independent task assignment. Unexpectedly, the Kanban boards also served as a platform for discussions about entry-specific details, and archiving such exchanges would have been valuable for preserving entry histories, similar to the “Discussion” pages used on Wikipedia (Supplementary Figs S21–S22). At the same time, issues arose when contributors self-assigned many more “stub entries” than they could possibly work on, despite guidelines to limit reservations. Further, contributors frequently forgot to unassign themselves from tasks, leading to confusion about task status. Nevertheless, the Kanban boards were welcomed by the participants, receiving a satisfaction score of 4.15/5.0 from questionnaire respondents (Supplementary File 3).

The MIBiG submission portal was a valuable upgrade from the previous spreadsheet-based approach. Structuring data submission through input forms encouraged participation without requiring prior training, while automated validation and the integrated review system reduced the incorporation of mis-annotated data. By the end of the Annotathons, ~40% of new or modified entries had successfully passed the review workflow, which was only possible due to the newly implemented infrastructure. Participants also expressed interest in a modular submission framework that would support annotation and review of specific data types in bulk, rather than entry by entry, enabling more effective use of specialist expertise (e.g. adding and reviewing of chemical structures). Although some contributors missed the simplicity of spreadsheets, overall satisfaction with the MIBiG submission portal was high, with an average satisfaction score of 4.16/5.0 (Supplementary File 3).

In comparison, respondents were less satisfied with the overall curation workflow, giving it an average score of 3.44/5.0 (Supplementary File 3). This was primarily due to the need to switch between separate platforms for coordination (Kanban) and data submission (submission portal). Additionally, the use of a “freemium” software solution for the Kanban board imposed limitations in availability, customizability, and required user consent to their terms of use (including the collection of personal data). Ideally, an open-source Kanban software would have been directly integrated into the submission portal, seamlessly linking task assignment with data curation and automatically managing entry reservations and task statuses. While this would enhance the user experience, developing such a sophisticated solution is labor-intensive, exceeds the capacities of most community initiatives, and raises concerns about long-term maintenance, funding, and sustainability [18]. One possible solution would be a crowd-sourced effort to develop a dedicated, free open-source software package that integrates task coordination, data submission and validation, and communication tools into a customizable curation framework, possibly as an extension of the existing Wikibase infrastructure (https://wikiba.se/). Examples for such reusable software packages are Canto [46] and Wikipathways [35], both providing infrastructure for various community-driven biocuration efforts [47, 49, 50]. The availability of such a software template would enable initiatives to focus their resources primarily on biocuration rather than platform engineering, thereby democratizing access. A “standardized” software package could also facilitate automated import of curated data into Wikidata, combining the strengths of specialized biocuration with the benefits of large-scale knowledge graph integration. While such an effort is not yet planned to originate from the MIBiG organizational team, we hope to contribute inspiration by making the source code of the MIBiG submission portal freely available [43].

In addition to technical workflows, the social workflows implemented in the MIBiG 4.0 curation model played a key role in facilitating the curation process. The assignment of predefined roles helped clarify responsibilities and improve communication. The core organizational team was instrumental in coordinating contributors, managing infrastructure, and resolving technical issues. The newly introduced roles of interest group coordinators (responsible for editorial oversight and clarifying edge cases) and reviewers (data quality assurance) were generally well-received, earning satisfaction scores of 3.8/5.0 and 4.0/5.0, respectively (Supplementary File 3). Notably, reviewers experienced a high workload, as they represented only 16.5% of participants. Future initiatives may benefit from investing in dedicated training to better define the scope of the review process and reduce the perceived workload. While clearly defined roles facilitate task triaging, smaller initiatives may struggle to recruit sufficient personnel. One possible solution could be consolidating roles like project and communication officer, or reviewer and interest group coordinator. Moreover, the sustainability and resilience of social workflows must be carefully considered. Redundancy should be built in to ensure continuity when key participants leave the project, and thorough documentation must be available to facilitate onboarding of new members. The establishment of a comprehensive governance model and a clear development roadmap is crucial to ensure the long-term success and sustainability of the project [18].

### Next steps and future perspectives

Since its inception a decade ago, the MIBiG database has been continuously expanded through regular annotation hackathons designed to keep pace with scientific advances. While the current community curation model enables efficient and timely participation, several aspects can still be improved. In preparation for the upcoming MIBiG 5.0 Annotathons, we plan to streamline our submission platform to integrate data curation and coordination, improve internal data validation and adherence to terminology, and to decrease our reliance on third-party tools. We will also consolidate the roles of interest group coordinators and reviewers into a unified “senior participant” role responsible for editorial oversight and data quality, with a target of ~25% of participants serving in this capacity to distribute workload more effectively and better support less-experienced contributors. In parallel, we are investigating a hybrid human-AI curation model, in which data parsing is performed by a domain-specific LLM and subsequently verified through expert review. This workflow has the potential to enhance the throughput of data curation by allocating human expertise to validation rather than data extraction. Furthermore, the prediction-validation feedback loop provides a foundation for active learning, enabling iterative model retraining through identification and correction of misannotations. Preliminary evaluations in our laboratories are promising (data not shown), but broader implementation of this approach is only envisioned for later iterations of the MIBiG Annotathons, once its robustness and workflow integration are more thoroughly assessed.

Despite the sustained efforts of the MIBiG community and other crowd-sourced curation initiatives, biocuration inherently lags behind the pace of primary research. This gap will persist until data deposition in machine-readable formats with rich metadata becomes a prerequisite for manuscript acceptance. To support this transition, MIBiG allows entries to be embargoed by the data submitter, enabling researchers to prepare and reference accession numbers during manuscript submission. In parallel, emerging publication formats such as micropublications [51] and nanopublications [52] are promising alternatives to traditional narrative scientific articles, allowing sharing of small, machine-readable knowledge snippets that are both citable and creditable. We are exploring ways to integrate such formats into MIBiG to enhance the recognition of individual contributions.

Ultimately, scientists generating primary data are best positioned to curate their results accurately. By engaging researchers directly and lowering the technical barriers to participation, initiatives such as MIBiG aim to promote a cultural shift in which biocuration becomes an integral and recognized component of the scientific publication process.

## Conclusions

In this work, we presented a new biocuration model, which was successfully applied to a large-scale, community-driven effort to annotate molecular data. By combining social and technical workflows, this cost-effective approach accommodates a high number of participants and appeals to both novice and expert contributors, creating opportunities for the former while making efficient use of the time of the latter. While the MIBiG curation model is particularly well suited to bioinformatics-based initiatives involving the extraction of gene, protein, and pathway annotation, chemical structure information, or phenotype association, its general principles (hackathon-centered community curation incentivized by co-authorship) are broadly applicable. Additionally, the MIBiG model fosters community-building and collaboration, which in turn drives a virtuous circle of data generation, curation, and reuse. These concepts can be extended to other biological and biomedical domains to promote community participation in data curation. The lessons learned from MIBiG 4.0 offer a blueprint for developing sustainable, open, and collaborative scientific resources that facilitate bioinformatics analyses and advance biological understanding.

**Key Points**

- Professional biocuration is essential for ensuring high-quality molecular datasets, but cannot keep pace with the rapid growth of biological data.
- Existing crowd-sourced strategies often lack scalability and rigorous data validation mechanisms.
- We present a new community-driven biocuration framework, developed during the MIBiG 4.0 annotation hackathons, which combines technical workflows with Annotathon-centered expert participation.
- This strategy enables the rapid generation of expert-curated molecular data and is transferable to other bioinformatics annotation initiatives.

## Supplementary Material

MIBiG_position_10_25_bib_revision_SI_bbaf659

Sup_1_MIBiG_4_initial_card_set_trello_NPI_bbaf659

Sup_2_edits_per_date_per_editor_bbaf659

Sup_3_MIBiG_4_0_Post_Annotathon_Questionnaire_NPI_bbaf659

## Data Availability

Computer code for the MIBiG submission portal web application is deposited on Zenodo under https://zenodo.org/records/13970328 [43].
