# Supplementary material for: Strategies for community-sourced biocuration in bioinformatics: a case study on MIBiG 4.0
Source: Brief Bioinform. 2025 Dec 11;26(6):bbaf659. doi: 10.1093/bib/bbaf659 (PMC12696713; doi:10.1093/bib/bbaf659)
Supplement: MIBiG_position_10_25_bib_revision_SI_bbaf659 [file mibig_position_10_25_bib_revision_si_bbaf659.pdf]

Strategies for community-sourced biocuration in bioinformatics: a case study on MIBiG 4.0 - Supplementary Information

Kai Blin[1], Catarina Loureiro[2], Nico L.L. Louwen[2], Jorge C. Navarro-Muñoz[2], Hans Gerstmans[3,4,5], Serina L. Robinson[6], Adriano Rutz[7], Zachary L. Reitz[8], Drew T. Doering[9], Justin J.J. van der Hooft[2,10], Tilmann Weber[1], Marnix H. Medema[2†] and Mitja M. Zdouc[2†]

[1]The Novo Nordisk Foundation Center for Biosustainability, Technical University of Denmark, Søltofts Plads, Building 220, 2800 Kongens Lyngby, Denmark
[2]Bioinformatics Group, Wageningen University & Research, Droevendaalsesteeg 1, 6708 PB Wageningen, the Netherlands
[3]VIB-KU Leuven Center for Microbiology, Flanders Institute for Biotechnology, Kasteelpark Arenberg 31, 3001 Leuven, Belgium
[4]Department of Biology, Laboratory for Biomolecular Discovery & Engineering, KU Leuven, Kasteelpark Arenberg 31, 3001 Leuven, Belgium
[5]Department of Biosystems, Biosensors Group, KU Leuven, Willem de Croylaan 42, box 2428, 3001 Leuven, Belgium
[6]Department of Environmental Microbiology, Swiss Federal Institute of Aquatic Science and Technology, Ueberlandstrasse 133, 8600 Duebendorf, Switzerland
[7]Institute for Molecular Systems Biology, ETH Zürich, Otto-Stern-Weg 3, 8093 Zürich, Switzerland
[8]Department of Ecology, Evolution and Marine Biology, University of California, 1169 Biological Sciences II, Santa Barbara, CA 93106, USA
[9]US Department of Energy Joint Genome Institute, Lawrence Berkeley National Laboratory, 1 Cyclotron Road, Berkeley, CA 94720, USA
[10]Department of Biochemistry, University of Johannesburg, C2 Lab Building 224, Kingsway Campus, Cnr University & Kingsway Road, Auckland Park, Johannesburg 2006, South Africa

[†] co-corresponding and co-last authors: M.H.M (email: marnix.medema@wur.nl, telephone: +31317482036), M.M.Z (email: mitja.zdouc@wur.nl, telephone: +31317482036)

Table of Contents

Strategies for community-sourced biocuration in bioinformatics: a case study on MIBiG 4.0 - Supplementary Information........1

Anonymized MIBiG 4.0 annotathon observer notes........ 3

Observer 1........ 3

Observer 2........ 6

Observer 3........ 7

Observer 4........ 8

Observer 5........ 9

Supplementary Figures........10

Anonymized MIBiG 4.0 annotathon observer notes

To provide a qualitative evaluation of the 2024 MIBiG 4.0 data curation events, five participants were enlisted to observe the annotathons and provide feedback.

Observer 1

Session 1 - 2024-03-29

- Trello board
  - So many of the cards had the ‘reserved’ tag, had to tell people to un-tag them unless they were ACTIVELY working on them
  - Accidentally archived at one point
- Reservations
  - Original idea was to save time — if people wanted to “work ahead”
  - But reserved cards were sitting idle, discouraging others from working on them, and that’s against the collaborative spirit
  - Going forward, don’t add the ‘reserved’ tag unless you REALLY know a lot about that BGC

Session 2 - 2024-04-23

- General
  - Existing record’s gene annotations differ from those in the paper, and NCBI doesn’t have any
  - Used liftoff tool to map from a similar BGC, resulting in better match with what authors reported
  - How to handle in the submission system?
  - Genome only available “on reasonable request to authors”
    - Option 1: Convince authors to upload the BGC to NCBI (and note that you emailed them on card)
    - Option 2: Use alternative strain that is known producer
    - If no known producer: Move to UNFIXABLE
- Reviewers
  - Two submissions (and cards) for same BGC
  - Do NOT delete either
  - Leave comment on one, port over any extra info, and move card to UNFIXABLE
- ig_nrps
  - New entry for compound already having an entry
  - If genuine duplicate: add comment “Duplicate of BGC____”, reviewer should not approve it, and move it to UNFIXABLE/CRITICAL ERROR board
  - If from different strain: OK to keep as its own entry
- ig_structures
  - Rigor of TLC→UV fluorescence as compound evidence (Aflatoxin BGCs)
  - Going forward, change retention time match with authentic standard → experimental values match with authentic standard

    - Potential novel compound class to add to the ontology
    - Existing records having swapped/incorrect names
- Identifiers (esp NCBI)
    - users unsure which DB to list for a cross-reference, submission UI only says when invalid
    - "Where do I find the GenBank ID of a protein?"
    - GOOD that there is a write-up on how to use IPG to get the GenBank-specific protein ID, just unfortunate that NCBIs DBs are complex
    - (Observation) Seeing the benefit of having controlled vocabularies, schemas, etc in "taming" the wide variation in the biology/chemistry, as well as user expectations/behavior
- ig_biological_activities
    - Users wanting to add more info about the NP's molecular/pathway target(s), but MIBiG is not focused on bioactivity
    - What to do about embargoed assemblies?
- Ig_exotic_bgcs
    - 2-3 BGCs in different locations contribute to production of 1 compound (arcuflavin)
    - Identification and Biosynthesis of a Novel Xanthomonadin-Dialkylresorcinol-Hybrid from Azoarcus sp. BH72
    - Marking first two as BGCs producing intermediates, then the final as a BGC producing the final
    - Also mark for future consideration
- GMT vs daylight savings (/winter time) confusion for Zoom start time

Session 3 - 2024-05-14

- Time zones tricky to communicate — "4-7pm GST+1 (British Summer Time)"
    - Solution: "Click this link to see this date/time converted to your time zone"
        - Calendar events (let the computer handle the conversion)
        - Caution: some sites/tools may have multiple listings for a given time zone that differ
        - https://time.is/
        - https://dateful.com/world-clock
- Looking like there will be a lot of "unfinished" entries — exotics, plants, … — what to prioritize? Many minimal entries / fewer complete entries?
- As a newcomer to the annotathons, collaboration has felt somewhat "scattered" — coordination of work for a single entry spans across:
    - Submission system - the actual submissions
    - Trello boards - the centralized workspace
    - Google Docs / YT - instructions
    - Slack - live / persistent chat, updated instructions
    - Zoom - live chat
    - Is there some other technology that integrates one/more of these (e.g. Slack + Trello boards to have a card-specific chatroom, similar to PR-specific channels for a GH-Slack integration)
    - GitHub "Projects", perhaps integrated with Slack?

Post-annotathon

- Many unfinished cards remain - counts from each list as of 2024-05-30:
    - 172 in "TO DO"

    - 161 in “IN PROGRESS”
    - 332 in “TO REVIEW”
    - 9 in “IN REVIEW”
    - 11 in “REVISION NEEDED”
    - 208 in “DONE”
    - 37 in “UNFIXABLE-CRITICAL ERROR”
- Colleagues who were busy during the annotathons noticed this, and wondered if it would help for them to become a reviewer now to help push these along.

Summary / General thoughts

- Coordination of this kind of work across so many people, time zones, fields of expertise, etc, is difficult. Having written instructions, video walkthroughs, the Slack workspace, and Zoom sessions were each helpful and important “pieces” to have, but to me at least, it still felt a bit hectic and like I needed to cross-reference many different windows on my computer – though perhaps that is because this was my first annotathon.
- I wonder if there are platforms where all these modes of communication/collaboration are more streamlined/integrated? Maybe migrating some of the how-to text from the Google Doc into tooltips/interactive guidance in the submission system?
- I envision something akin to GitHub issues and pull requests, where updates are reviewed prior to merging and each issue/PR has its own discussion thread that can be linked to other related ones if mentioned. Totally acknowledge that this is a complex problem!
- Clearly there remains a ton of BGC data to finish curating – I think the “rolling release” model mentioned in an email is a great idea so that these data can make their way onto MIBiG without having to wait for a yearly cycle.
- Data accessions and identifiers can be a difficult concept to grasp for people unfamiliar with (relational) databases or with the “layout” of NCBI’s databases. For example, there are many different things that a “GenBank ID” can refer to: a protein, an assembly, a nucleotide record, a gene, etc. (NCBI’s documentation is extensive but much of it is old and scattered). This would be a big undertaking (and probably out of scope for MIBiG) if it doesn’t already exist, but it’d be nice to have something akin to UniProt’s ID Mapping service/API, but for NCBI’s databases and accessions, and that is better or even just simpler than the EDirect API.

Observer 2

- Cautionary tale about reliance on free but not open source software (Trello). Created some hiccups when things changed (user limit, potential loss of editing capabilities)
- submission platform very under-tested.
- Coordinators and reviewers should've tested it by annotating an entry themselves at least a month prior to first session
- Looking forward, it would be very valuable to have an overview of all the missing datapoints (or issues) for each entry, a pool of publications for people to start new entries, better de-replication (e.g. same organism and metabolite? perhaps part of existing entry), a pool of publications with problems ("unfixable", but could become useful if problems solved in another publication)
- Would be great if some things in the platform were interactive (e.g. select domain to assign specificity, automatically run some things like interpro scan to get base functional annotation, tools to improve gene structure annotations)
- Observation: even if 400 people signed up for it, only a fraction interacted in Slack (in the main channels). Did they see the answers to the questions being put there?
- Observation: some people claimed a ton of entries, but still after the third annotathon they are still on the to-do list (perhaps limit the amount of simultaneous entries people can work on)
- Sometimes a consensus is needed for some things (e.g. "we need really hard evidence" vs "protein identity == 100% in related organism is enough" or "this entry should be retired/unretired"). This is hard to communicate (and actually hard to get the consensus; and who is making these decisions?). Do we need a governance model?
- I sense a lack of reviewers. Will see how this affects finishing the remaining entries.
- kanban principle useful but need to define scope of each card. Some people took very seriously the call to have "maximum entries" and kicked back entries that were not fully complete. But different people have different scopes so perhaps the cards should be somewhat "atomized". Do we have the minimal entry? is it correct? then there are these related cards detailing annotation of more in-depth data
- No good approach yet to avoid duplication of data (i.e. DOIs are never checked to see if they already appear in an existing or in-progress entry)
- Sometimes hard to keep up with answers in Slack. Solutions to common problems

Observer 3

Thank you so much for the email and preparing the nice survey which I just filled out. I think the survey was very well written to assess how things went overall.

- The annotathon was in general extremely well-organized, especially considering the immensity of the team involved. Kudos to you for finding a way to bring order to the Chaos!
- The submission portal helped to standardize everything but the combination with Trello created some inefficiencies as I was constantly flipping back-and-forth between the two and it felt often like I was doing things twice. This is certainly a case of my own unfamiliarity with the Kanban principles and the system - in the future I think it would improve with experience, but I also would support a move away from Trello (which I think you were planning to do anyways giving all the login/licensing issues)
- Overall myself and multiple people I spoke to felt less efficient somehow in the whole process as compared to early annotations. I found myself struggling down many rabbit holes which was unexpected given that the MIBiG 2.0 annotation was far less structured yet felt more efficient. It felt somehow 'ok' to have an incomplete or minimal entry submitted whereas here with the submission I felt compelled to fill out all the criteria and therefore took much longer per entry than last year. If the goal is more completeness I think (hope) the submission portal achieved this goal.
- The part which was really lacking this time was the social connection. The last annotathon from MIBiG 2.0 (redacted) and I described as 'cozy' which turned into a really nice networking opportunity as there were at least 5-6 of us in a breakout room with cameras on chatting here and there, which hardly ever happened this time. Most of the breakout rooms had 0-1 people in it and nearly all had their cameras off the whole time, at least from the breakout rooms that I was in. Maybe this was just circumstantial.
- The Slack was fantastic for resolving issues in real-time e..g, the issue with NPAtlas inconsistencies etc.

Observer 4

Before the start of the Annotathon

- Example Annotations: As someone new to the annotation process, it would be incredibly beneficial to have a completely filled-out example annotation available for review before or during the annotathon sessions. This example would serve as a practical guide, demonstrating where to find necessary information and how to properly input data. I understand that preparing such an example would require considerable effort from the organizers, but it could significantly enhance the learning curve for new participants.
- Pre-annotathon Training Sessions: Consider organizing brief training sessions or webinars before the annotathon begins. These sessions could cover basic annotation techniques, introduce the tools that will be used, and answer any preliminary questions participants might have.
- BGC Reservations: Reserving Biosynthetic Gene Clusters (BGCs) should continue to be allowed, but perhaps only if the individual or group has previously published the relevant BGC, structure, etc. This information could be included on the reservation card to ensure clarity.
- Glossary of Terms: There are instances where multiple labels can be applied to the same structure or modification. Creating a general dictionary or glossary to explain these terms would be highly useful in resolving any ambiguities.

During the Annotathon

- Communication Platforms: Slack has proven to be an excellent tool for posting questions and resolving issues quickly. However, it appears that some questions, particularly those related to the submission portal or general queries, were asked multiple times. An overview of frequently resolved issues could be maintained to address this redundancy.
- Trello Board Efficiency: The Trello board used for managing entries was highly effective and should definitely be retained for future annotathons. However, there were instances where it was unclear whether participants had signed off from a card, making it difficult to ascertain the status of their work. Implementing a system to indicate whether an entry is still actively being worked on could help mitigate this issue.
- Coordinator Engagement: The activity and engagement of RiPP coordinators during each session were commendable. However, during one session, participants from other groups joined, likely due to the visible activity, but their questions were often too specific to be answered by the present coordinators. It might be beneficial to have backup coordinators available for each group or to more clearly define the coordinators' expected roles and responsibilities.
- Adapting Existing MIBiG Entries: When modifying existing MIBiG entries, it was not always clear what needed to be changed. In general, more clarity is needed regarding the scope of required adaptations. Providing more detailed guidelines on what to check and adapt would be helpful.
- Reviewing entries: In general entries stay long in the “TO REVIEW” or “IN REVIEW” column. If a document or guideline could be made on how to check the new entries, maybe more people would feel comfortable to also review new entries.

Observer 5

Comments regarding Kanban board:

- "Fixes needed" was where cards went to die. Some people (mostly names I knew) would correct their mistakes when tagged, but I think many had already left by the time their entry was reviewed.
- Annotators frequently put entries missing entire sections in the "to be reviewed" category. I would review what was there, and then send it back to "in progress" so that someone could add the missing sections. Other reviewers put them in "fixes needed" (as instructed), but they would then sit there because annotators didn't know to look there.
- As a reviewer, it would have been nice to see which sections were already reviewed in the portal.
- I think that the tags on the cards should be TO-DOs, like "needs tailoring" or "needs structure", so that people who are only interested in a single task can easily find what needs to be done

In all, I thought the card system was an improvement, but this really seems to need a custom solution. I think treating each section as a separate subtask under an umbrella entry would help a lot.

Supplementary Figures

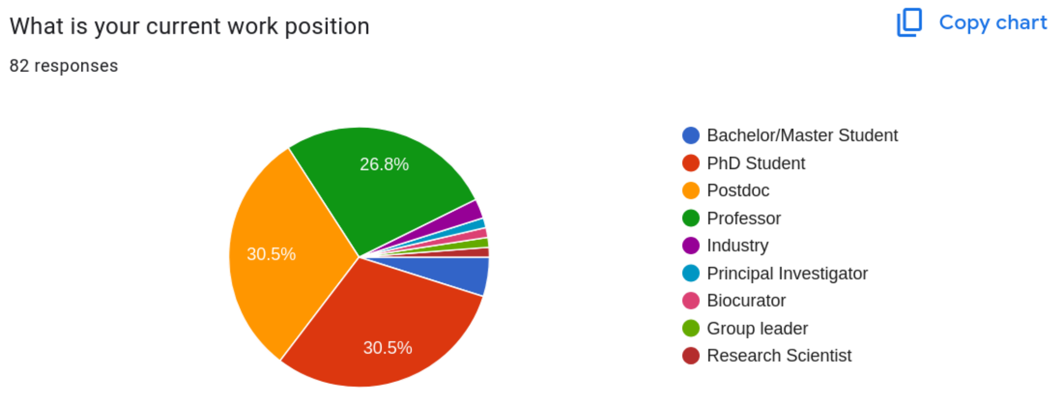

Figure S1: Pie-chart of participant's current work position.

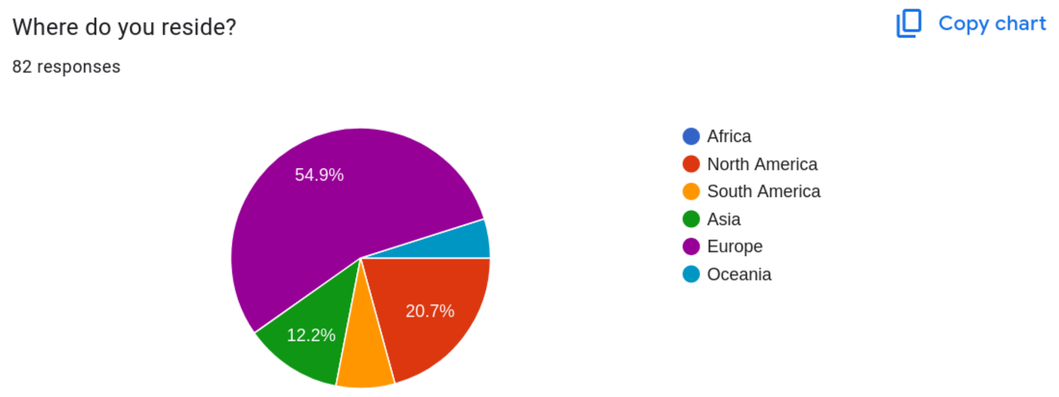

Figure S2: Pie-chart of residence.

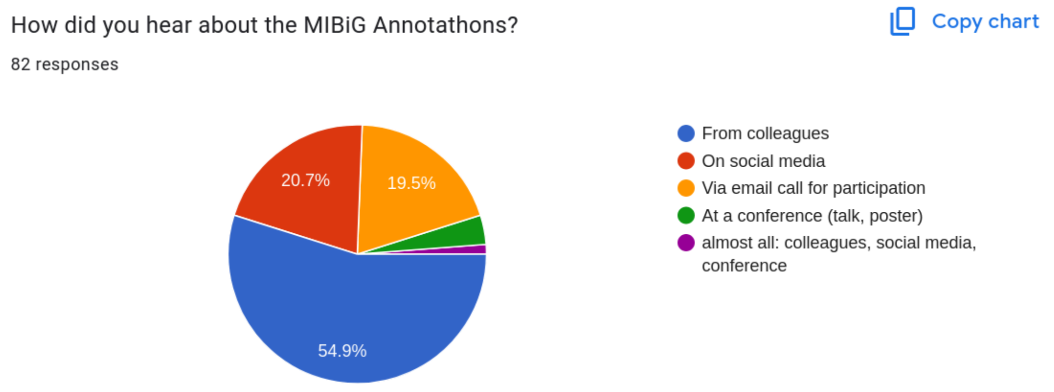

Figure S3: Pie-chart of where the participant had heard of the MIBiG Annotathons.

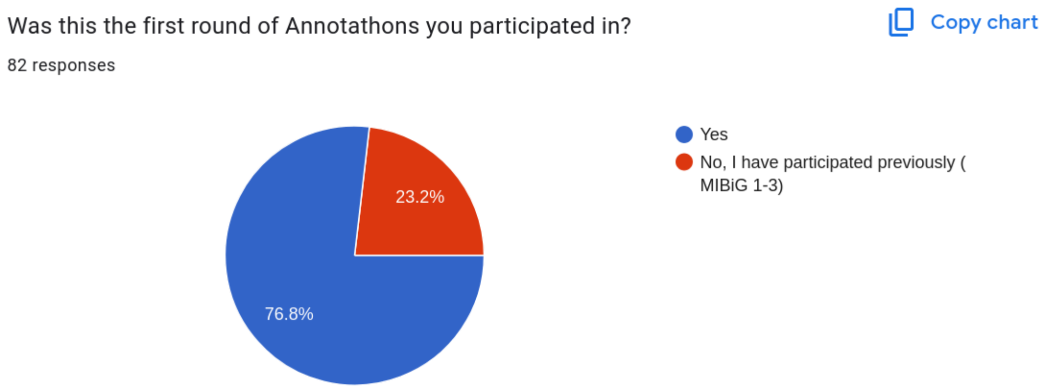

Figure S4: Pie-chart of first-time participation.

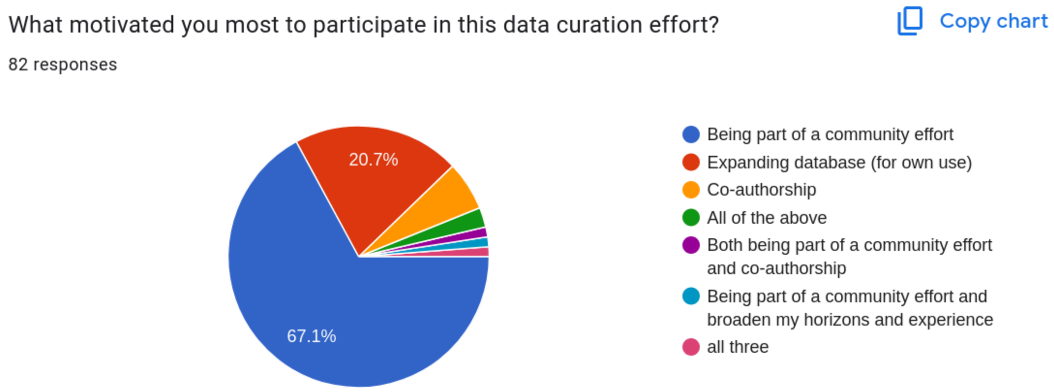

Figure S5: Pie-chart of main motivation for participation.

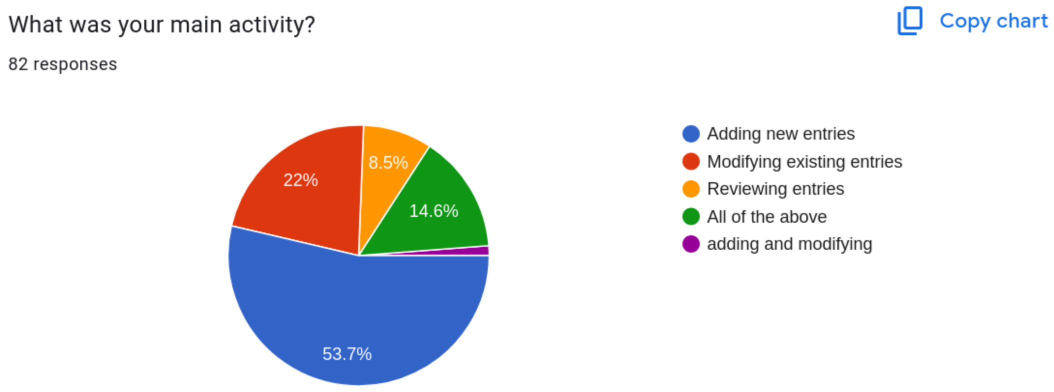

Figure S6: Pie-chart of main activity of participant.

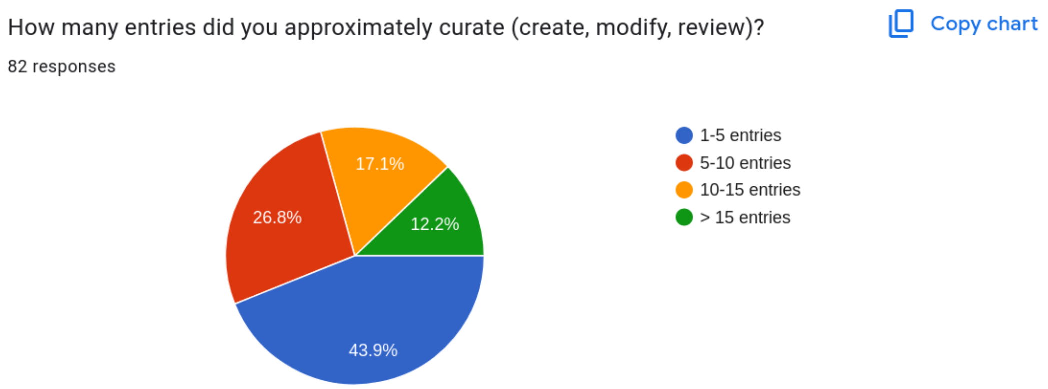

Figure S7: Pie-chart of number of entries curated.

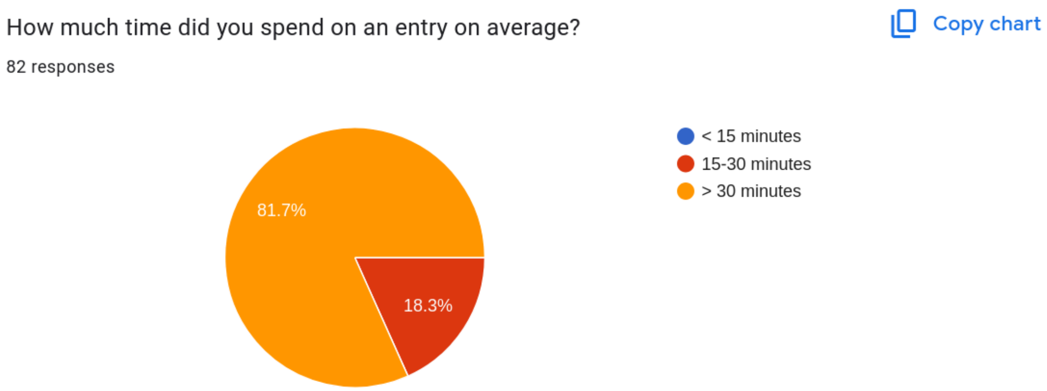

Figure S8: Pie-chart of time spent on curation per entry.

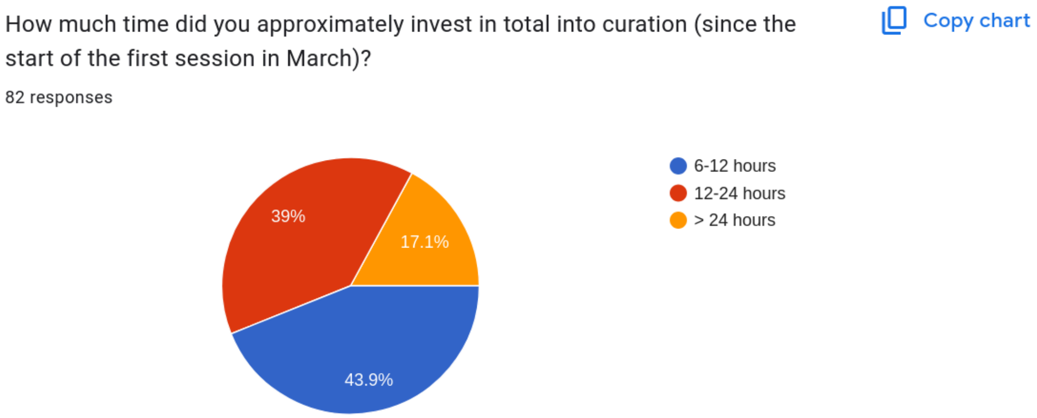

Figure S9: Pie-chart of total time invested in curation.

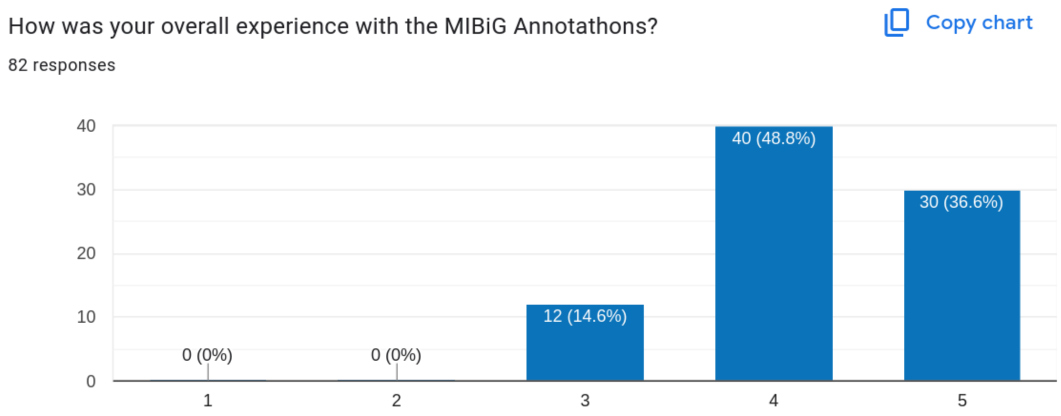

Figure S10: Bar-chart of overall experience (5=best, 1=worst).

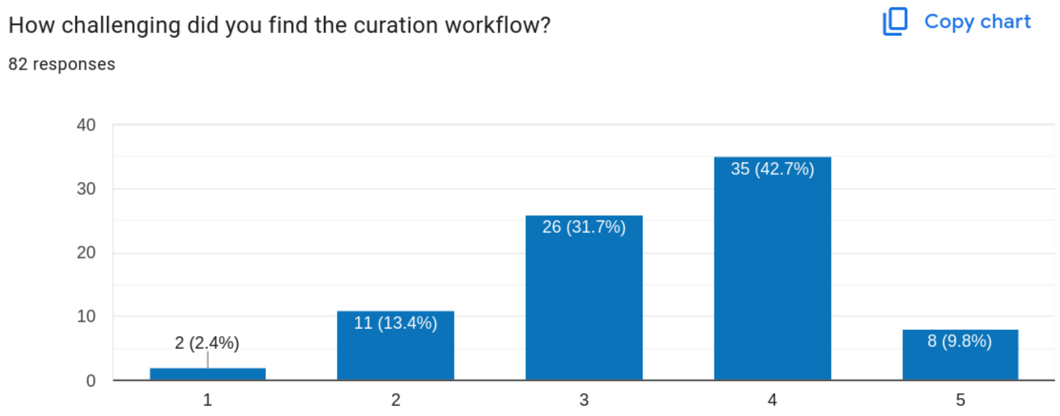

Figure S11: Bar-chart of satisfaction with curation workflow (5=easy, 1=hard).

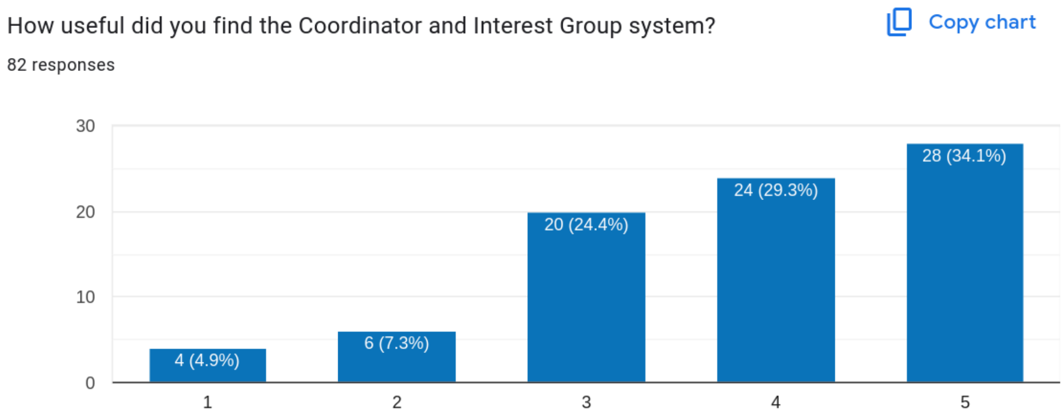

Figure S12: Bar-chart of satisfaction with coordinator and interest group system (5=very satisfied, 1=not satisfied).

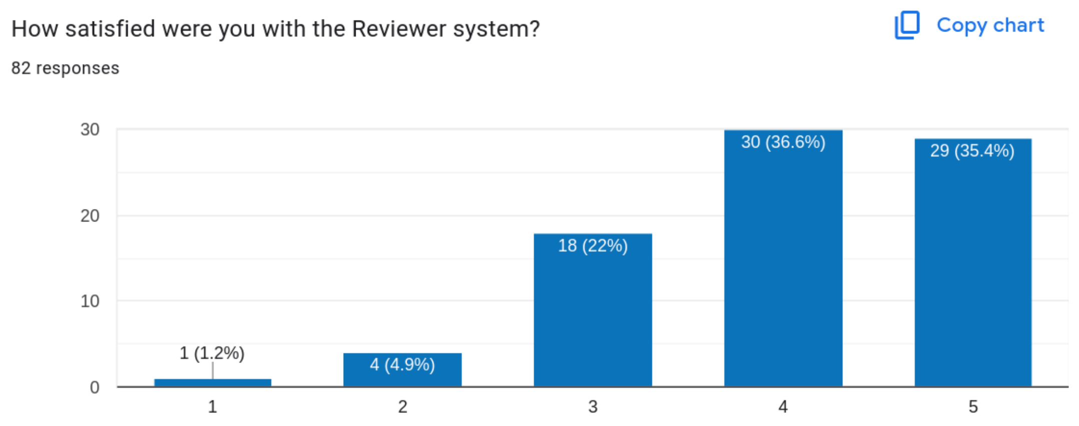

Figure S13: Bar-chart of satisfaction with reviewer system (5=very satisfied, 1=not satisfied).

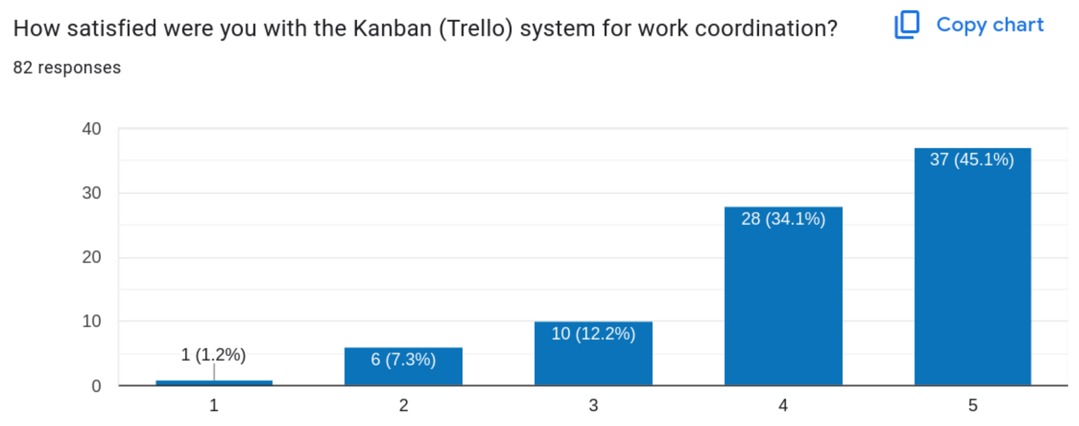

Figure S14: Bar-chart of satisfaction with Kanban system for work coordinator (5=very satisfied, 1=not satisfied).

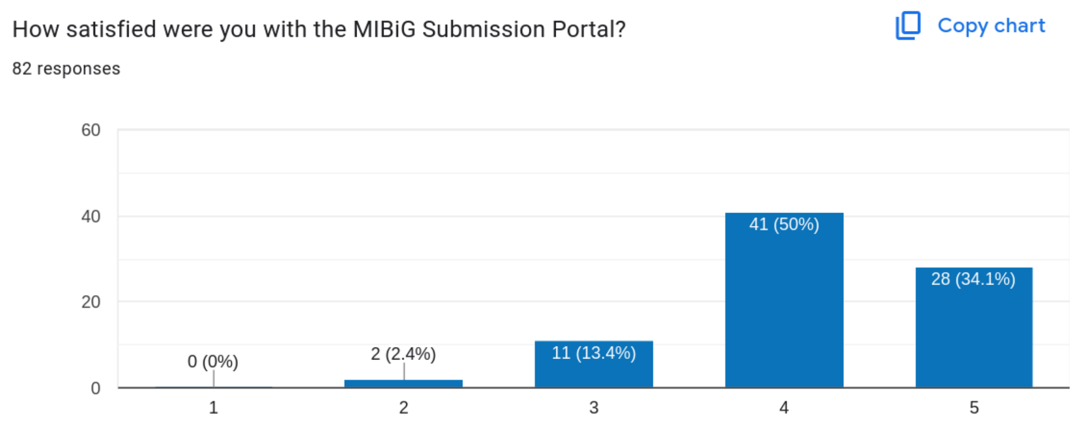

Figure S15: Bar-chart of satisfaction with MIBiG submission portal (5=very satisfied, 1=not satisfied).

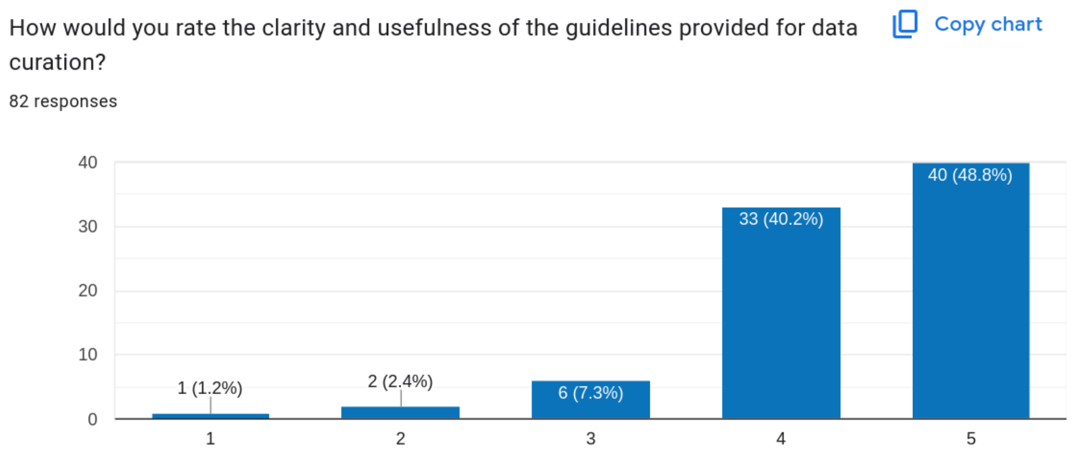

Figure S16: Bar-chart of satisfaction with training materials (5=very satisfied, 1=not satisfied).

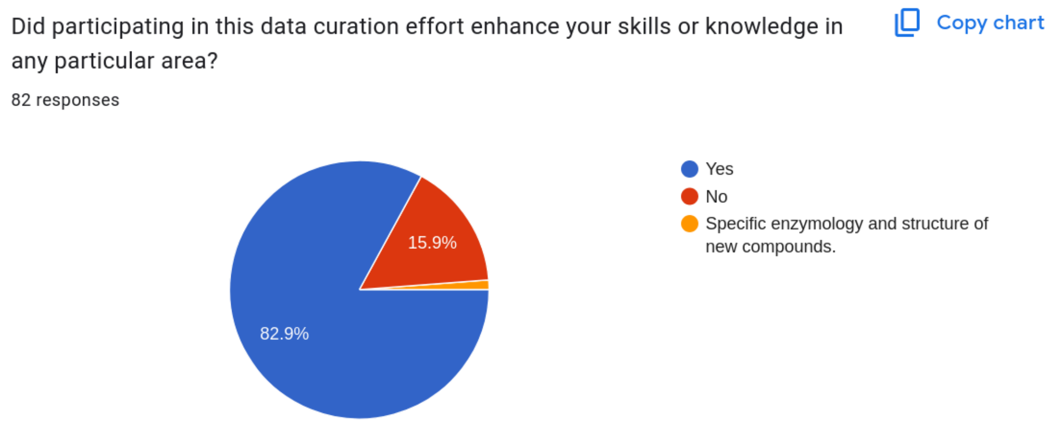

Figure S17: Pie-chart of skill gain.

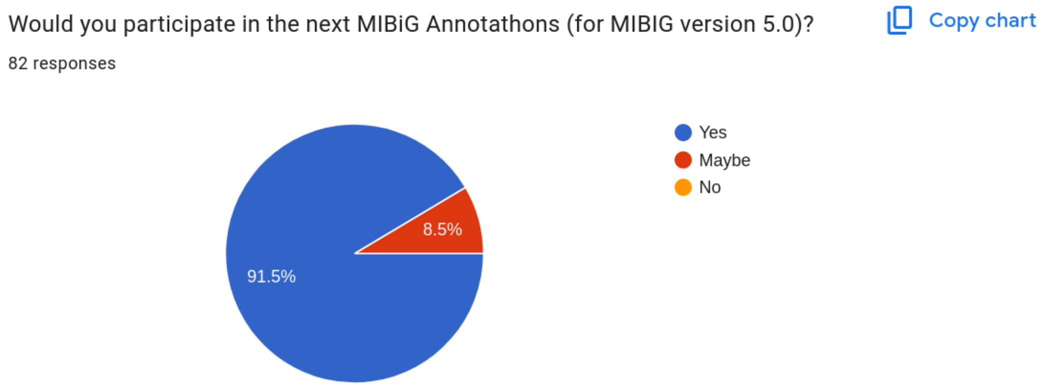

Figure S18: Pie-chart of participation in next iteration of MIBiG curation hackathons.

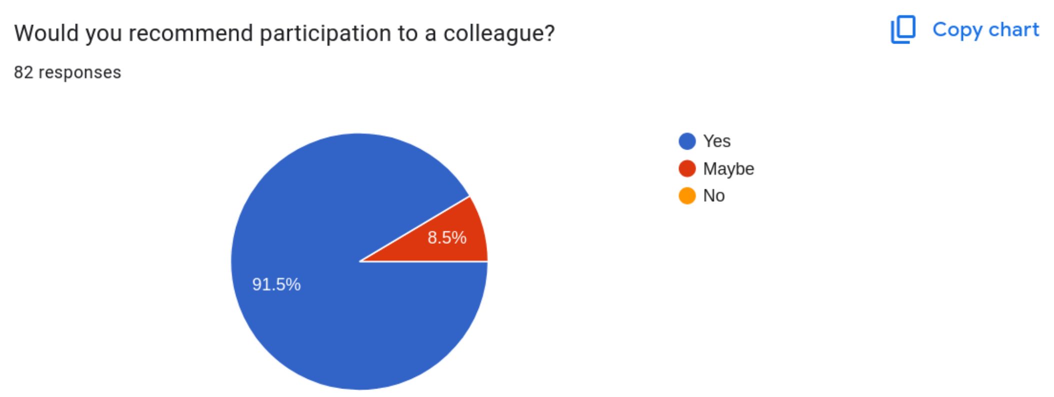

Figure S19: Pie-chart of planned recommendation to a colleague.

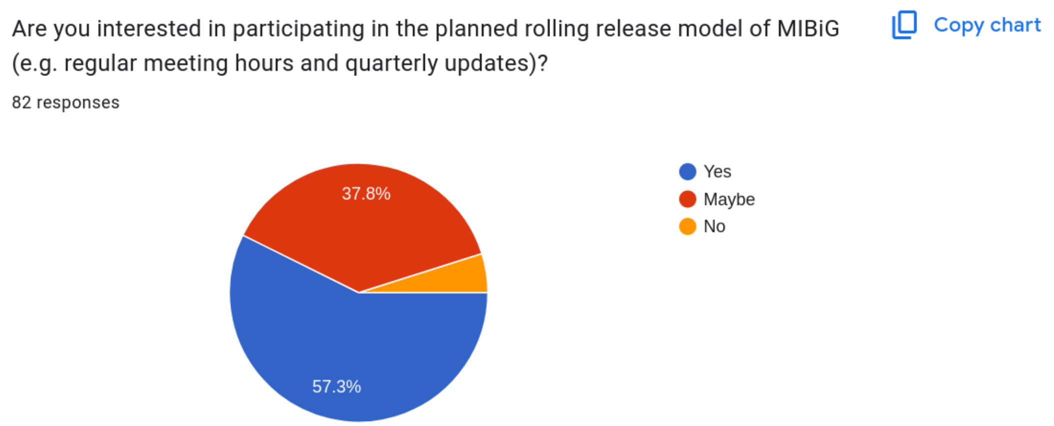

Figure S20: Pie-chart of interest in rolling release system.

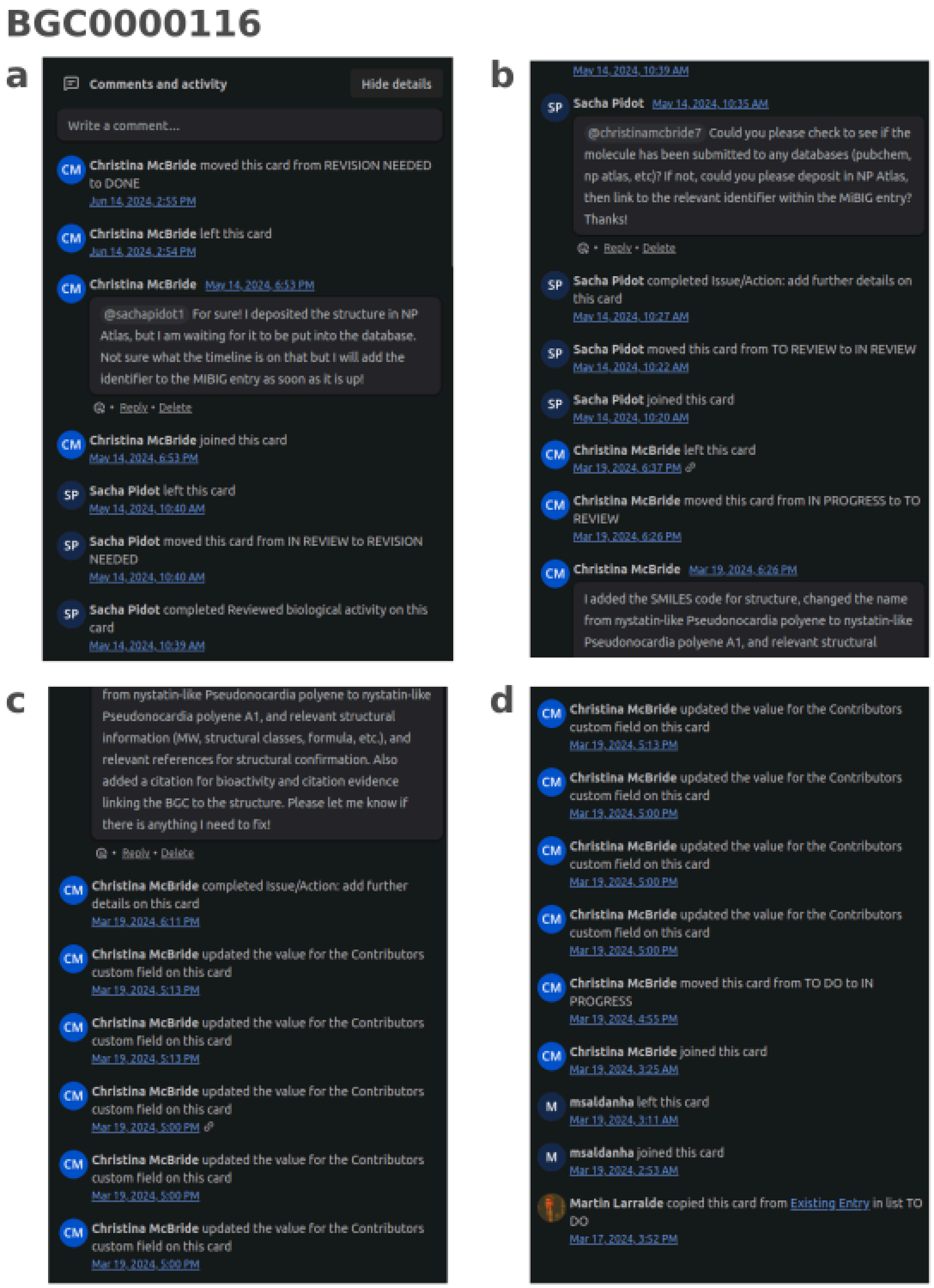

Figure S21: Editing history of Kanban card for BGC0000116. Note the reverse-chronological order a-d.

Epoxyquinomicin b

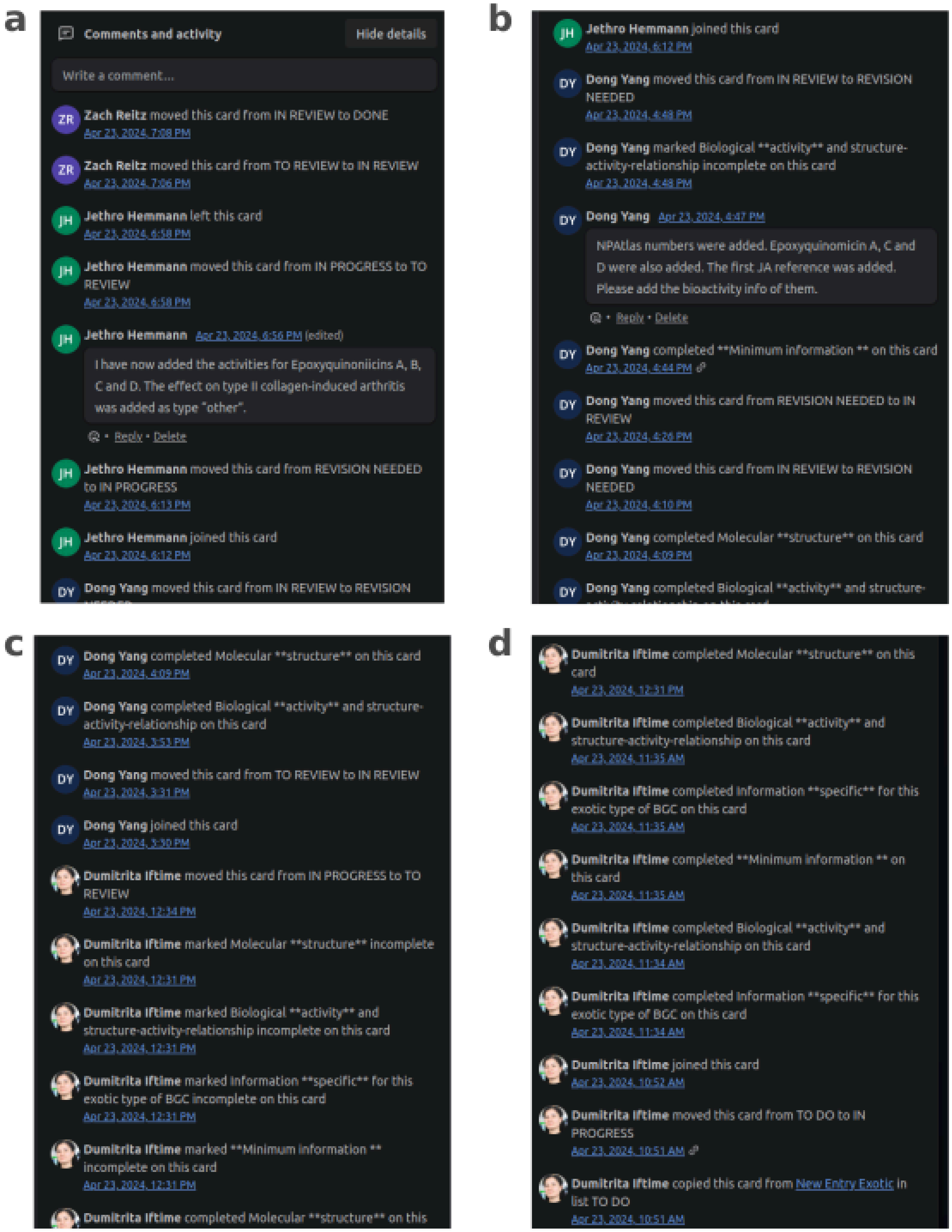

Figure S22: Editing history of Kanban card for the new entry epoxyquinomicin b . Note the reverse-chronological order a-d.
